# Correction: *Fractional flow reserve derived from microcatheters versus standard pressure wires: a stenosis-level meta-analysis*

**DOI:** 10.1136/openhrt-2018-000971corr1

**Published:** 2019-07-17

Seligman H, Shun-Shin MJ, Vasireddy A, *et al*. Fractional flow reserve derived from microcatheters versus standard pressure wires: a stenosis-level meta-analysis. *Open Heart* 2019;6:e000971. doi: 10.1136/openhrt-2018-000971

The license type of the paper has changed from CC BY-NC to CC BY.

